# Effect of intake of selected nutrients on skin firmness and elasticity in women

**DOI:** 10.3389/fnut.2024.1483678

**Published:** 2024-11-11

**Authors:** Aleksandra Podgórska, Aleksandra Kicman, Sylwia Naliwajko, Marta Wacewicz-Muczyńska, Marek Niczyporuk

**Affiliations:** ^1^Department of Aesthetic Medicine, Medical University of Bialystok, Białystok, Poland; ^2^Department of Bromatology, Medical University of Bialystok, Białystok, Poland; ^3^Department of Specialist Cosmetology, Medical University of Bialystok, Białystok, Poland

**Keywords:** skin elasticity, skin firmness, skin hydration, sebum, nutrients, diet, courage + Khazaka test

## Abstract

**Introduction:**

The proper functioning of the skin is influenced by a proper diet. The purpose of this study was to determine the effect of selected nutrients on selected skin parameters.

**Methods:**

The study group included 59 women, who were divided into two age groups (under and over 40  years old). A three-day 24-h dietary interview was conducted among the study women and skin parameters were assessed using the Courage + Khazaka method.

**Results:**

The results showed LA, ALA, Dietary fiber, Na, K, Mg, Zn, Cu, Vitamin E, Thiamine, Riboflavin, Folates, Vitamins B6, B12 and C correlated with skin elasticity. On the other hand, skin elasticity is influenced exclusively by vitamin D acted on skin firmness.

**Conclusion:**

The results obtained indicate that a diet rich in appropriate nutrients has a positive effect on the proper maintenance of important skin parameters.

## 1 Introduction

The skin is the largest organ in the human body and complex biological structure, consisting of three basic layers such as the epidermis, dermis and subcutaneous tissue (1–3). It is an organ with many important functions such as a physical barrier, demarcating deeper tissues from the external environment, while providing its contact through perception of stimuli or exchange of substances (1, 3, 4). The skin also protects against biological agents and, due to its acidic pH, prevents pathogens from penetrating deep into the skin (5–7). In addition, it provides protection against chemical agents in the form of, among others, corrosive, irritating or allergenic substances on the skin, as well as physical agents such as ionizing radiation, infrared radiation and sunlight (3, 6). The skin also plays an important role in maintaining water and electrolyte balance, immune response and thermoregulation (1, 8). Due to the presence of numerous nerve endings and mechano-receptors, it is also an important sensory organ (1, 3). In order to effectively perform all of the above-mentioned functions, the skin must be properly hydrated and the hydro-lipidic layer of proper composition (9).

The condition of the skin is influenced by many factors, both environmental and genetic (e.g., diet, cigarettes, alcohol or weather conditions), which can cause oxidative-reductive disorders. An inadequate diet can compromise the integrity of the skin and impair its biological function (9–11). Foods, therefore, can exhibit not only positive but also negative effects on skin health and condition (12, 13). A properly balanced diet should contain ingredients from all categories, that is, essential nutrients, vitamins and minerals, supplied in the amounts recommended by experts. In addition, it should focus on the supply of adequate fluids (8, 9).

Highly specialized measuring devices, which do not disturb the integrity of the skin layers, are now used to assess skin condition. Their precision, sensitivity or repeatability make them extremely useful. With their help, many important skin parameters can be measured, such as the degree of hydration and lubrication, elasticity, pH or evaporation (14).

Therefore, the purpose of this study was to evaluate the effect of selected nutrients on the level of elasticity and firmness of women’s skin.

## 2 Materials and methods

### 2.1 Patients

Fifty-nine women between the ages of 21 and 74 participated in the study. The women were divided into two age groups: under (*n* = 37) and over (*n* = 22) 40 years old (Table 1). This division was based on the goal of testing whether the skin condition of women in both groups is mainly influenced by diet or also or exclusively by processes occurring not only in their skin, but the entire body, such as age-related hormonal changes. Their height ranged from 157 to 188 cm, and weight between 45 and 95 kg. The women’s BMI showed underweight to first-degree obesity. Characteristics of the studied groups with determined statistical parameters can be found in Table 1. Women were selected based on the criterion of dietary support in the form of dietary supplements. Only women with no supplementation were included in the study. In addition, women with chronic diseases or skin diseases who were undergoing drug therapy were excluded from the study. Women who were on a specialized diet at the time of the study, aimed at achieving specific effects by them, were also excluded from the study. The women also underwent cosmetic interviews with a cosmetologist prior to inclusion in the study, who evaluated their existing home care. When he detected abnormalities, appropriate changes were made to make the care appropriate for the skin type. These changes, however, took place only in the area of basic care, and excluded the use of preparations that significantly affect the condition of the skin, such as exfoliating or vitamin preparations. In addition, the probands did not use any of the treatments in both cosmetology and esthetic medicine during the study. All women participating in the study gave written consent for the analysis. The study received approval from the Bioethics Committee of the Medical University of Bialystok number: APK.002.428.2021.

**Table 1:** Characteristics of the study group (*n* = 59).

Parameters	Av. ± SD	Min-Max	Med.	Q1–Q3
Age (years)	37 ± 14	21–74	32	25–48
Age < 40 (*n* = 37)	28 ± 5	21–40	27	24–32
Age > 40 (*n* = 22)	52 ± 9	40–74	49	47–60
Height (cm)	167 ± 6	157–188	167	164–171
Weight (kg)	64 ± 11	45–95	62	57–69
BMI (kg/m^2^)	22.8 ± 3.8	15.9–34.2	21.6	20.2–24.1

Av., average; Max, maximum; Med., median; Min, minimum; SD, standard deviation; Q1, lower quartile; Q3, upper quartile.

### 2.2 Preparing the skin for the test

The women rested for about 10–15 min before taking skin measurements. During this time, the test skin area, which was the forehead, was, if makeup or skin care products were present, cleansed with micellar lotion, then gently rinsed with water and dried with a disposable towel. Measurements were taken under ambient conditions of about 20°C and humidity ranging from 40 to 60%.

### 2.3 Testing of skin firmness and elasticity

A specialized Cutometer® dual MPA 580 measuring device (Courage + Khazaka Electronic, Köln, Germany) was used to test the skin. It is a scientific multi-probe system for measuring skin properties. It consists of a basic device (base) and a set of selected measurement probes from the world leader in the production of skin testing equipment Courage + Khazaka, for professional measurement of not only skin parameters, but also hair. The system allows the connection of probes to measure, among others, skin elasticity (Cutometer®), skin surface hydration (Corneometer® CM 825), transepidermal water loss (Tewameter® TM Hex), skin pH (Skin pH-Meter PH 905) or sebum content (Sebumeter® SM 815) (15).

Measurements were made by using a probe for determining the viscoelastic properties of the skin (Cutometer®). Cutometer® is used to measure the elasticity of the upper layer of the skin using vacuum, which mechanically deforms the skin. The measurement is based on the suction method. The device, generating a vacuum, pulls the skin into a small probe slot and then releases it after a specified time. Inside the probe, using a noncontact optical measurement system, an assessment is made of the depth of penetration, according to which the intensity of light in the system varies. The skin’s resistance to the vacuum produced by the device—firmness—and its ability to return to its original state—elasticity—are displayed in the form of curves during real-time measurements. Based on these, a number of parameters that determine the elastic and viscoelastic properties of the skin surface are calculated. However, the obtained results can be quite often variable due to numerous factors, i.e., tissue heterogeneity, the amount of skin in the probe by applying suction, operator variability or maintenance of the probe and device (15, 16).

In the present study, we evaluated the parameters, i.e., R0, R1, R2, R5 and R7 (17–19) as shown in Table 2. The choice of these parameters was dictated by the information provided by the manufacturer of the device, especially since these parameters are the most important in assessing elasticity and provide the most objective results. In addition, these were the parameters most often described in other studies conducted using Courage & Khazaka, addressing the topic as skin testing.

**Table 2:** Characteristic of parameters of skin firmness and elasticity.

Parameter	Characteristics
R0 [mm]	Amplitude at the end of the suction phase of the first curve Reflects the firmness/flexibility of the skin The lower its value is, the better the skin firmness is
R1 [mm]	The amplitude at the end of the relaxation time of the first curve The ability of the skin to return to its resting state The smaller its value, the better the skin shows the ability to return
R2 [%]	The ratio of the amplitude after suction and the ability to return to the resting state Total skin elasticity The higher its value is (closer to 1 = 100%), the better the skin elasticity is
R5 [%]	Net elasticity The higher its value is (closer to 1 = 100%), the better the skin elasticity is
R7 [%]	Immediate regeneration within the first 0.1 s compared to the amplitude after aspiration The higher its value is (closer to 1 = 100%), the better the skin elasticity is

### 2.4 Estimating intake of selected nutrients

A three-day 24-h dietary interview (2 weekdays+1 weekend day) recommended by the Committee on Human Nutrition Sciences of the Polish Academy of Sciences in Warsaw was conducted among the women surveyed. The interviews were analyzed using the computer program Diet 6.0. developed at the Independent Laboratory of Epidemiology and Nutrition Standards of the Food and Nutrition Institute in 2018 in Warsaw. This program provides the ability to calculate all nutrients, i.e., calories, proteins, carbohydrates, fats, as well as vitamins, micronutrients or individual fatty acids and others. The obtained values were compared with Polish standards (20), the values of which are given in Table 3, comparable to the reference values of nutrients issued by the European Food Safety Authority (EFSA) (21).

**Table 3:** Dietary reference values of nutrients for women.

Alimentary component	EFSA recommendation	Nutrition standards for the Polish population
LA (% of energy) [g]	4% of energy	4% of energy (AI = 10.2 g)
ALA (% of energy) [g]	0.5% of energy	0.5% of energy (AI =1.3 g)
Dietary fiber [g]	AI = 25	AI = 25
Na [mg]	AI = 2000	AI = 1,500
K [mg]	PRI = 3,500	AI = 3,500
Mg [mg]	PRI = 300	EAR = 255
Zn [mg]	AR = 6.2	EAR = 6.8
Cu [mg]	PRI = 1.3	EAR = 0.7
Vitamin A [μg RE]	AR = 490	EAR = 500
Vitamin E [mg α-TE]	PRI = 11	AI = 8
Vitamin D [μg]	PRI = 15	AI = 15
Vitamin B_1_ [mg]	AR = 0.7 (0.072 mg/MJ)	EAR = 0.9
Vitamin B_2_ [mg]	AR = 1.3	EAR = 0.9
Vitamin B_6_ [mg]	AR = 1.3	EAR = 1.1
Vitamin B_12_ [μg]	PRI = 4	EAR = 2.0
Vitamin C [mg]	AR = 80	EAR = 60
Folates [μg DFE]	AI = 330	EAR = 320

AI, adequate intake; ALA, alpha-linolenic acid; AR, Average Requirement; α-TE, α-tocopherol equivalent; Cu, copper; DFE, dietary folate equivalent; EAR, Estimated Average Requirement; EFSA, European Food Safety Authority; K, potassium; LA, linoleic acid; Mg, magnesium; Na, sodium; PRI, Population Reference Intakes; RE, retinol equivalent; Zn, zinc.

### 2.5 Statistical analysis

The data obtained were analyzed using Statistica V 13.3. statistical program and Microsoft Office Excel 2019 software. K - S and Lilliefors and W Shapiro–Wilk test was used to assess the normality of the data distribution. The nonparametric distribution of the data distribution was shown. Parameters of descriptive statistics were also calculated, i.e., mean, median, minimum, maximum, lower AND upper quartile and standard deviation. Spearman’s R test was used to assess correlation, dividing the data into two groups: < 40 and > 40 years. R represents the strength and direction of the association between two ranking variables. The level of statistical significance was taken as *p* < 0.05.

## 3 Results

### 3.1 Characteristics of skin firmness and elasticity parameters

It was shown that the values reflecting firmness (R0) and the ability of the skin to return to its resting state after the skin suction phase (R1) did not differ between the groups under and over 40 years of age. The median of the R0 parameter for the <40 and > 40 years groups, respectively, was 0.26 mm and 0.21 mm, while the R1 parameter was the same in both groups, i.e., 0.07 mm. Statistically significant differences were found in the other three parameters, i.e., total skin elasticity (R2), net elasticity (R5) and immediate recovery within the first 0.1 s compared to the amplitude after aspiration (R7). In each of these parameters, higher median values were shown in the under 40 group, indicating better skin elasticity in women in this age group. The exact figures are shown in Table 4.

**Table 4:** Descriptive characteristics of skin firmness and elasticity parameters using the specialized Courage-Khazaka measuring device.

Parameter	Av. ± SD (Min.-Max.)	Med. (Q1-Q3)	Dependence
R0 [mm] Age <40 vs. > 40	0.24 ± 0.10 (0.08–0.73)	0.24 (0.18–0.30)	Smaller value = higher firmness
	Av. 0.25 ± 0.08 vs. 0.23 ± 0.13 Med. 0.26 vs. 0.21		
R1 [mm] Age < 40 vs. > 40	0.07 ± 0.03 (0.02–0.19)	0.07 (0.05–0.09)	Smaller value = higher firmness
	Av. 0.07 ± 0.03 vs. 0.07 ± 0.03 Med. 0.07 vs. 0.07		
R2 [%] Age < 40 vs. > 40	71.04 ± 10.69 (43.40–117.55)	69.55 (65.05–77.30)	Higher value = higher elasticity
	Av. 73.48 ± 11.43 vs. 66.94 ± 7.97 Med. 71.30 vs. 68.88*, *p < 0.02		
R5 [%] Age < 40 vs. > 40	64.08 ± 13.05 (19.15–89.25)	63.80 (55.25–72.75)	Higher value = higher elasticity
	Av. 69.01 ± 11.16 vs. 55.78 ± 11.93 Med. 70.05 vs. 56.33*, *p < 0.001		
R7 [%] Age < 40 vs. > 40	41.24 ± 8.71 (13.80–60.20)	40.15 (35.60–47.70)	Higher value = higher elasticity
	Av. 44.97 ± 7.88 vs. 34.98 ± 6.14 Med. 44.95 vs. 36.70*, *p < 0.001		

AI, adequate intake; ALA, alpha-linolenic acid; AR, Average Requirement; α-TE, α-tocopherol equivalent; Cu, copper; DFE, dietary folate equivalent; EAR, Estimated Average Requirement; EFSA, European Food Safety Authority; K, potassium; LA, linoleic acid; Mg, magnesium; Na, sodium; PRI, Population Reference Intakes; RE, retinol equivalent; Zn, zinc.

### 3.2 Intake of selected nutrients with diet

The results of the intake of selected nutrients with diet are shown in Table 5. The percentage of subjects with sufficient (above median of AI) and insufficient (below median of EAR) intake was evaluated. No significant statistical differences were observed between the intake of selected nutrients according to the age of the women studied.

**Table 5:** Selected nutrients in the diet.

	Consumption		Median of norm	Sufficient consumption [n (%)]	Insufficient consumption [n (%)]
Alimentary component	Av. ± SD Min-Max	Med. Q1–Q3			
LA [g]	6.7 ± 3.8 2.8–19.2	5.2 4.0–7.9	AI = 10.2	9 (15.3%)	–
ALA [g]	1.1 ± 0.5 0.4–2.87	0.9 0.7–1.3	AI = 1.3	16 (27,1%)	–
Dietary fiber [g]	16.26 ± 6.7 6.1–39.8	16.1 11.2–18.8	AI = 25	5 (8.5%)	–
Na [mg]	2555.4 ± 786.1 1073.2–4600.5	2445.0 2030.4–2967.6	AI = 1,500	54 (91.5%)	–
K [mg]	2396.6 ± 826.1 1228.0–5102.6	2260.8 1806.9–2928.4	AI = 3,500	5 (8.5%)	–
Mg [mg]	253.8 ± 96.0 126.4–527.2	223.3 190.3–308.9	EAR = 255	–	38 (64.4%)
Zn [mg]	8.2 ± 2.5 4.1–15.1	7.8 6.1–10.2	EAR = 6.8	–	20 (33.9%)
Cu [mg]	1.0 ± 0.4 0.4–2.3	0.9 0.7–1.3	EAR = 0.7	–	14 (23.7%)
Vitamin A [μg RE]	1107.3 ± 1474.5 226.7–11391.6	823.9 592.7–1140.3	EAR = 500	–	8 (13.6%)
Vitamin E [mg α-TE]	8.8 ± 4.8 3.6–26.0	6.8 5.2–11.5	AI = 8	23 (39.0%)	–
Vitamin D [μg]	3.8 ± 6.8 0.3–51.0	2.2 1.3–3.7	AI = 15	1 (1.7%)	–
Vitamin B_1_ [mg]	0.9 ± 0.3 0.4–1.8	0.9 0.7–1.1	EAR = 0.9	–	31 (52.5%)
Vitamin B_2_ [mg]	1.4 ± 0.7 0.6–5.4	1.3 1.1–1.7	EAR = 0.9	–	3 (5.1%)
Vitamin B_6_ [mg]	1.6 ± 0.9 0.7–6.2	1.4 1.1–1.9	EAR = 1.1	–	12 (20.3%)
Vitamin B_12_ [μg]	3.9 ± 5.7 0.7–45.6	2.8 2.3–3.6	EAR = 2.0	–	5 (8.5%)
Vitamin C	108.6 ± 95.1 16.4–558.6	96.9 54.1–129.4	EAR = 60	–	20 (33.9%)
Folates [μg DFE]	250.6 ± 146.2 1112.4–1074.6	210.3 175.5–275.5	EAR = 320	–	50 (84.7%)

AI, adequate intake; ALA, α-linoleic acid; α-TE, α-tocopherol equivalent; Av., average; Cu, copper; DFE, dietary folate equivalent; EAR, estimated average requirement; K, potassium; LA, linoleic acid; Max., maximum; Med., median; Mg, magnesium; Min., minimum; Na, sodium; RE, retinol equivalent; SD, standard deviation; Q1, lower quartil; Q3, upper quartil; Zn, zinc.

### 3.3 Correlations between intake of selected nutrients and skin firmness and elasticity

Correlations are summarized in Table 6. By age group (< 40 and >40 years old), statistically significant correlations were found only in the group of women <40 years old, as indicated in the Table 6.

**Table 6:** Correlations between intake of selected nutrients and skin firmness and elasticity (in the table are only statistically significant correlations, *p* < 0.05).

Skin parameter alimentary component	Firmness R0	Elasticity			
		R1	R2	R5	R7
LA	x	x	0.408^*^	0.289 0.337^*^	0.267 0.347^*^
ALA	x	x	0.303 0.533^*^	0.367 0.530^*^	0.386 0.517^*^
Dietary fiber	x	−0.306	0.356 0.421^*^	x	X
Na	x	x	x	−0.319	−0.300
K	x	−0.325	0.328 0.329^*^	x	X
Mg	x	−0.287	0.391 0.462^*^	x	X
Zn	x	x	0.285 0.330^*^	x	X
Cu	x	−0.364	0.328 0.424^*^	0.262	X
Vitamin A	x	x	0.327^*^	x	X
Vitamin D	−0.273	x	x	x	X
Vitamin E	x	−0.281	0.267 0.531^*^	0.305 0.433^*^	0.364^*^
Vitamin B_1_	x	x	0.325 0.581^*^	x	0.435^*^
Vitamin B_2_	x	−0.273	0.258	x	X
Vitamin B_6_	x	x	0.338 0.381^*^	x	0.360^*^
Vitamin B_12_	x	−0.261	x	x	X
Vitamin C	x	x	0.311 0.506^*^	x	0.342^*^
Folates	x	−0.304	0.326 0.494^*^	x	X

x, lack of correlation; *, correlation in the age group < 40; ALA, α-linoleic acid; Cu, copper; K, potassium; LA, linoleic acid; Mg, magnesium; Na, sodium; Zn, zinc.

**Figure 1:**
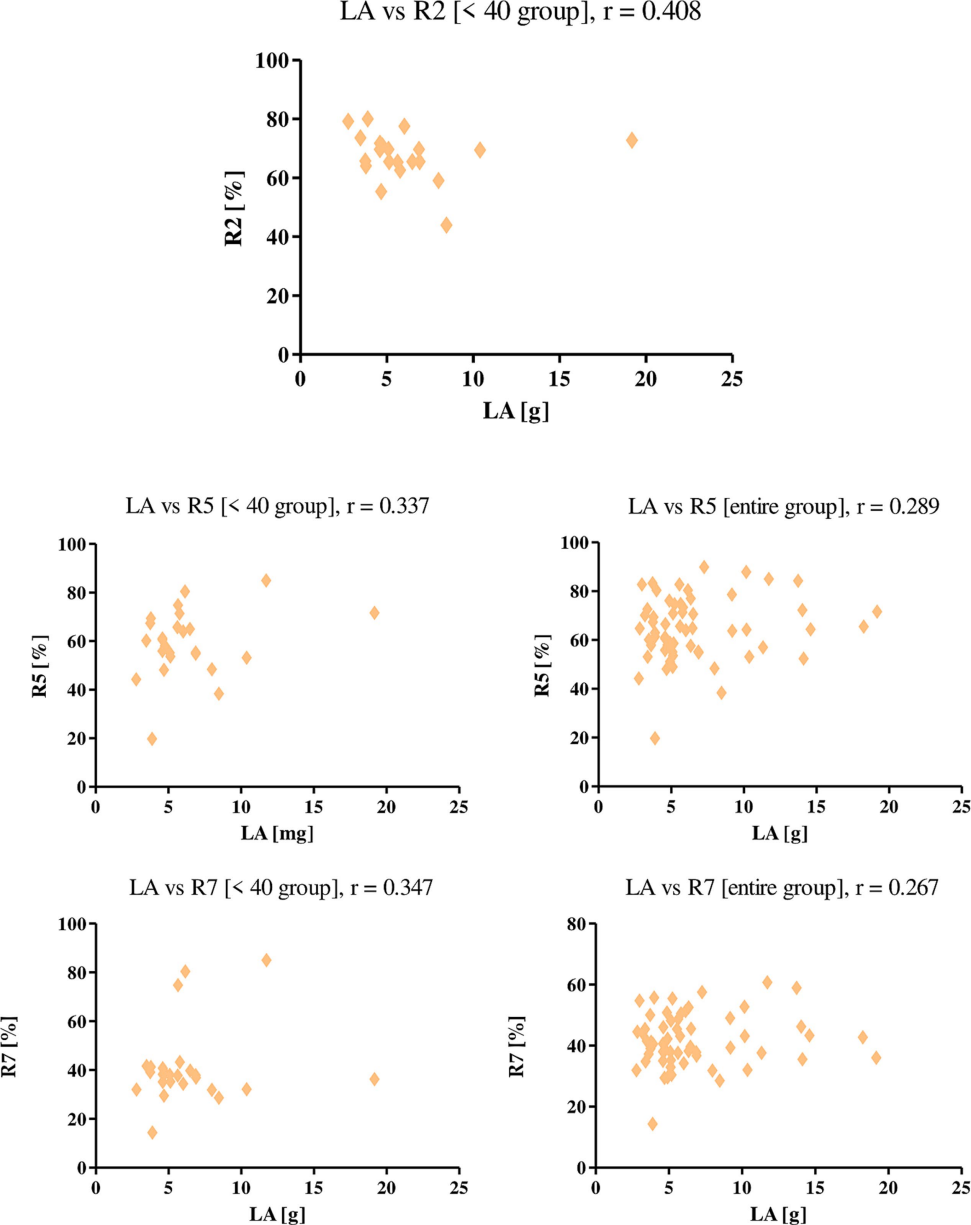
Correlations for linoleic acid, with a reported *r* value.

Linolenic acid intake with diet correlated positively with R2, R5 and R7 parameters (Figure 1). Correlations were shown in both the entire study group and the <40 age group for parameters R5 and R7, the values were 0.289, 0.267 for the total group and 0.337, 0.347 for the <40 age group, respectively. Linolenic acid intake correlated with parameter R2 only in the group of women under 40, and was 0.408. This means that the higher the intake of the acid with the diet, the higher the level of skin elasticity.

**Figure 2:**
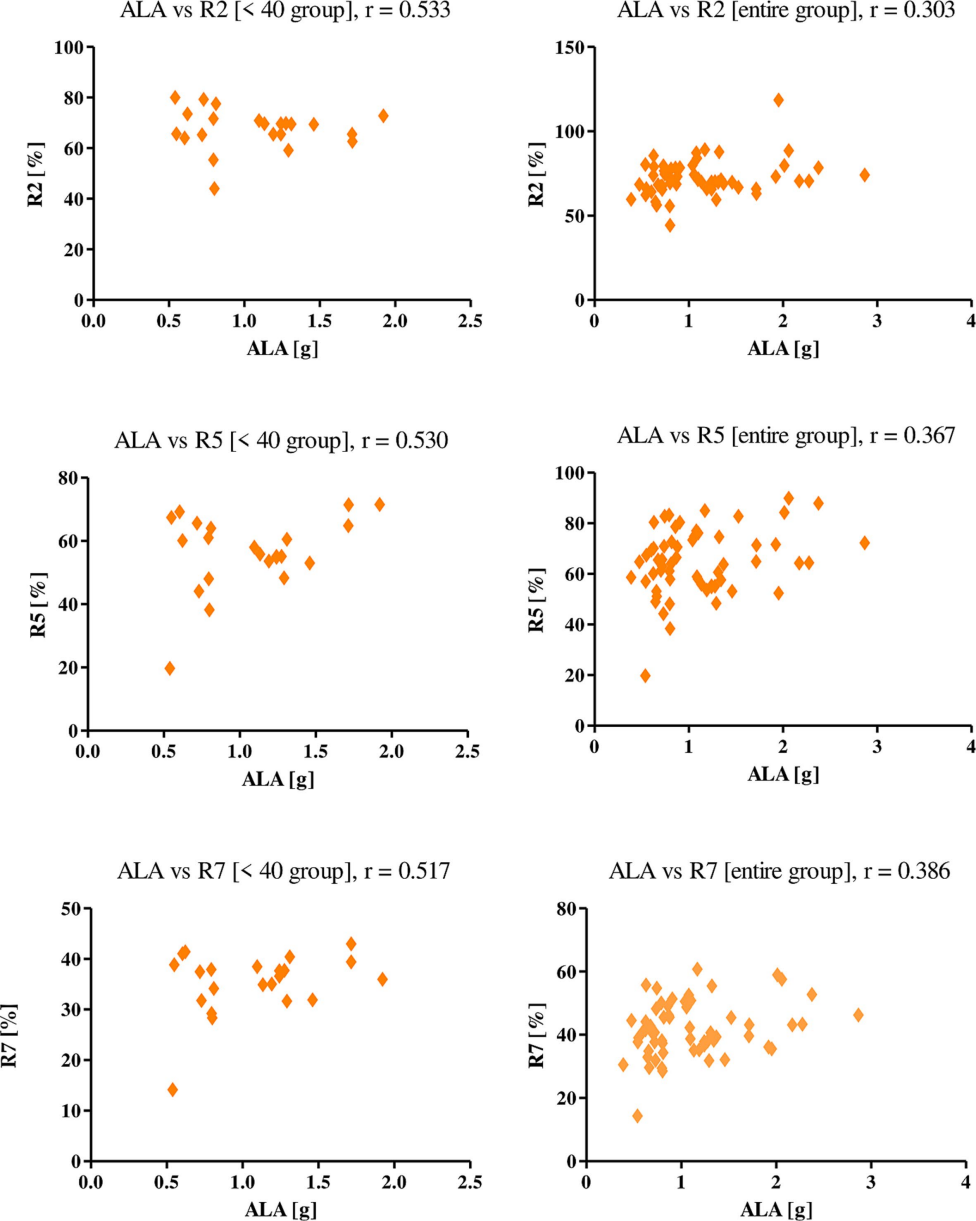
Correlations for *α*-linoleic acid with a reported *r* value.

A positive correlation was also shown for the intake of alpha-linolenic acid, indicating its positive effect on the skin elasticity of the women studied. Correlations were presented with the parameters R2, R5 and R7, both in the entire study group and among women under 40 years of age (Figure 2). The values for R2, R5 and R7, respectively, among all the female subjects were 0.303, 0.367, 0.386, while those of women in the <40 group were as follows: 0.533, 0.530, and 0.517.

**Figure 3:**
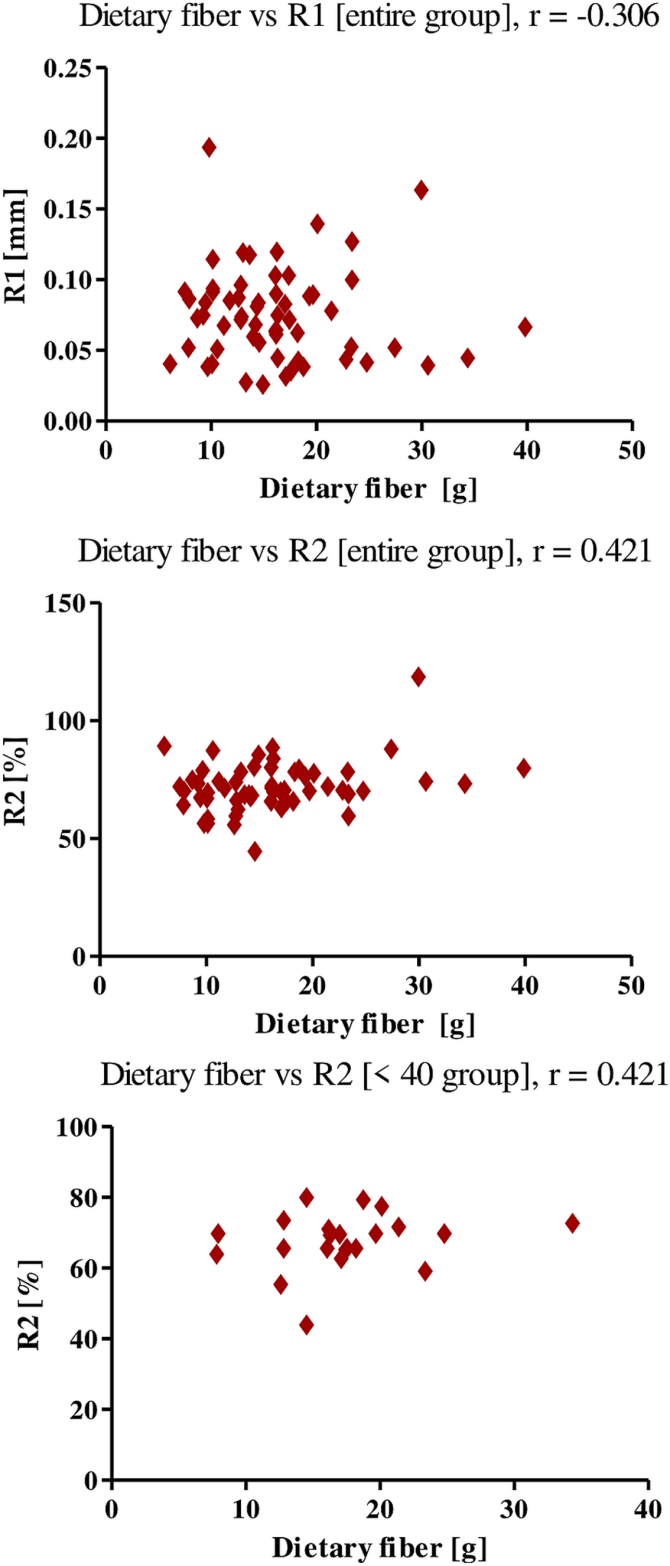
Correlations for dietary fiber with a reported *r* value.

Dietary fiber showed a negative correlation with the R1 parameter for the group as a whole, and a positive correlation with the R2 parameter, both in the entire study group and in the <40 group, which means that higher dietary fiber intake negatively affects the firmness of women’s skin, while positively affects its elasticity. The *r-value* for R1 was −0.306, for R2 0.356 (entire study group) and 0.421 (age group <40; Figure 3).

**Figure 4:**
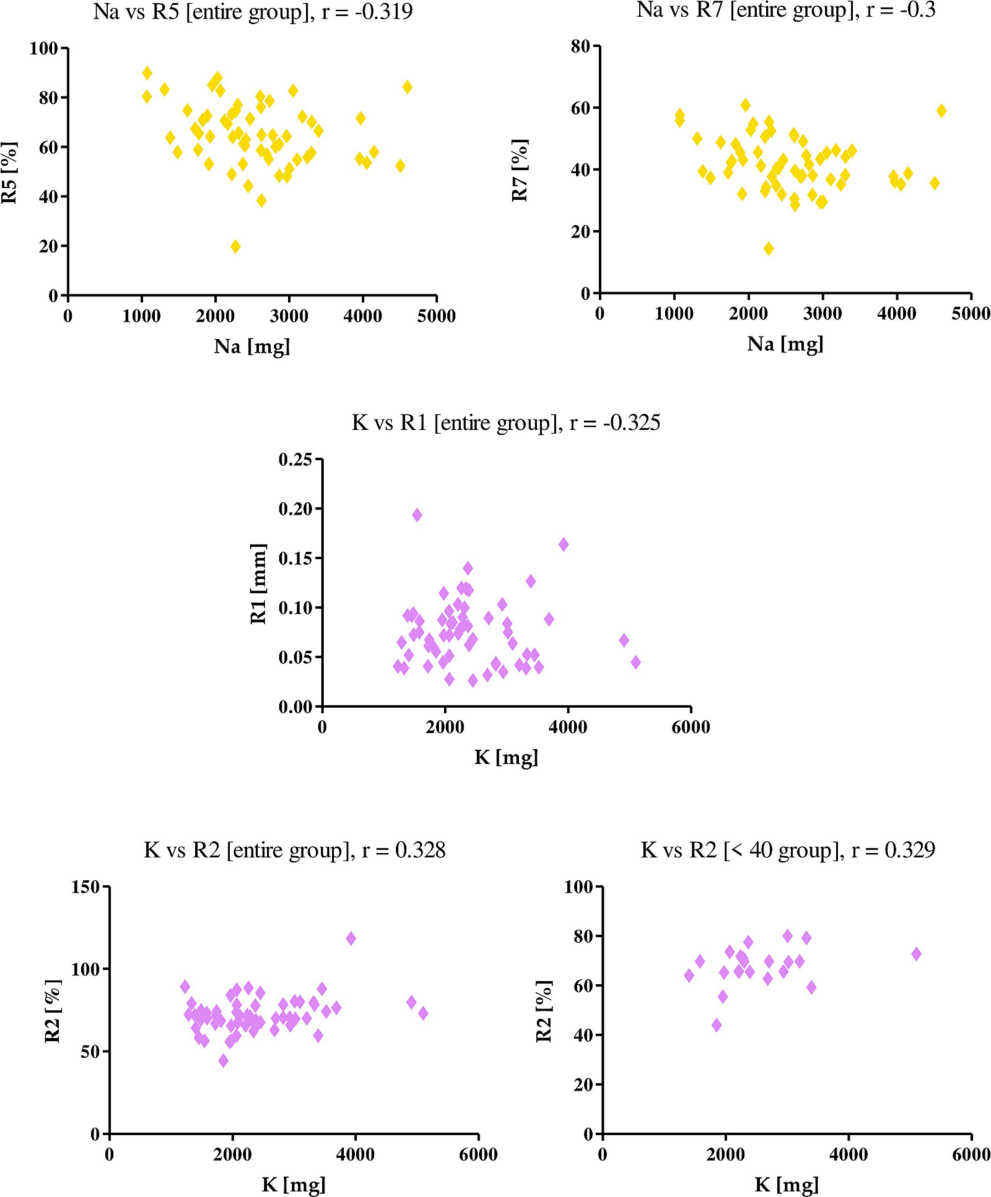
Correlations for sodium and potassium with a reported *r* value.

Sodium and potassium intake with diet correlated in skin firmness and elasticity parameters (Figure 4). Sodium showed a negative correlation with parameters R5 (*r* = −0.319), R7 (*r* = −0.300) among the entire study group, indicating that higher sodium intake negatively affects skin elasticity. Potassium negatively correlated with the R1 parameter in the group of female subjects, where r was −0.325, while it positively correlated with R2, considering both the entire study group and the <40 age group, where r was 0.328, 0.329, respectively. This means that as potassium intake increases with diet, skin firmness decreases, while skin elasticity increases.

**Figure 5:**
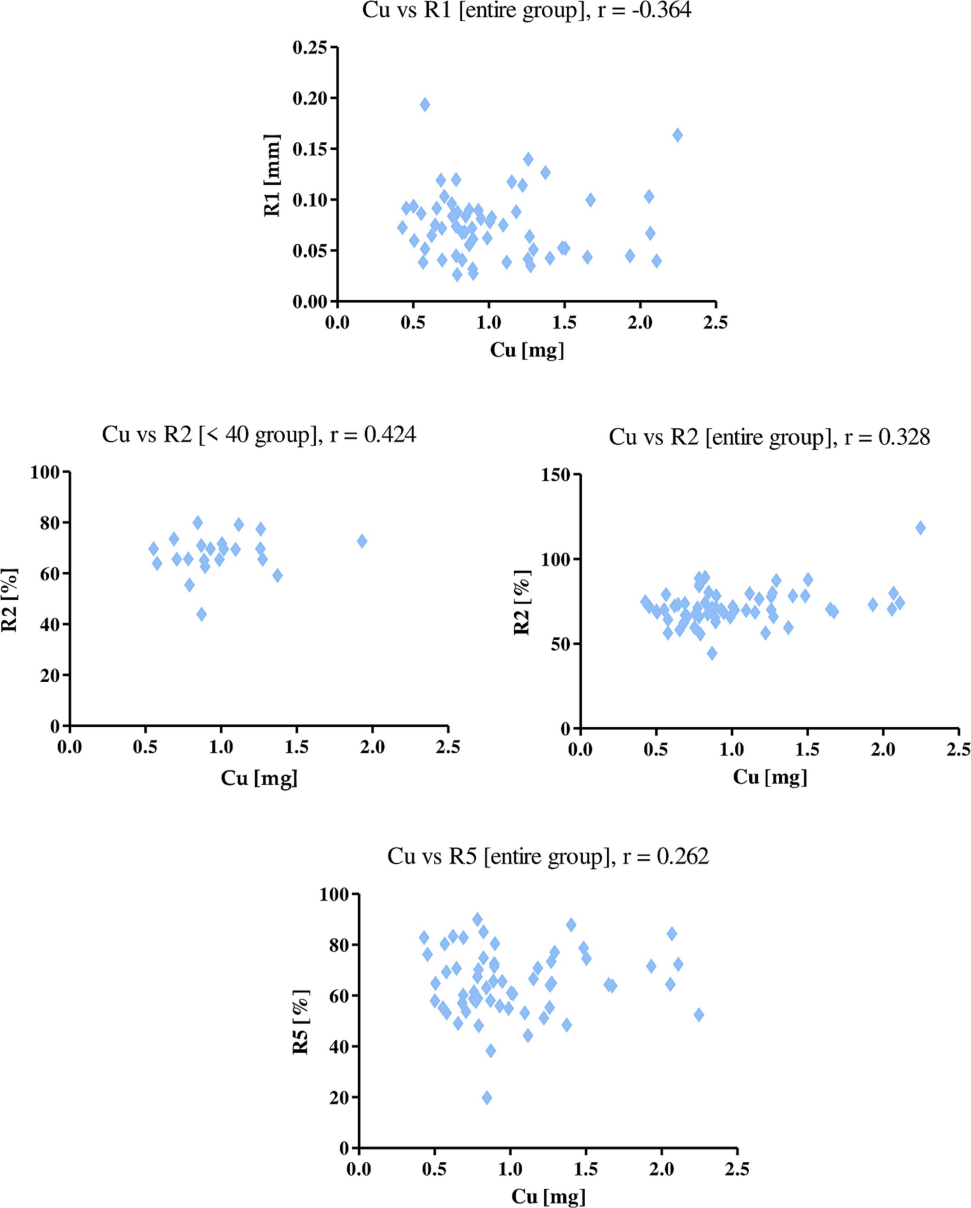
Correlations for magnesium and zinc with a reported *r* value.

Magnesium showed a negative correlation with the R1 parameter (*r* = −0.287), indicating a negative effect of dietary magnesium on skin firmness. With the R2 parameter, magnesium showed a positive correlation in both the entire study group and the group of women under 40. The r value was 0.391 and 0.462, respectively. This correlation indicates that magnesium has a positive effect on the elasticity of women’s skin. A similar relationship was shown for zinc, which positively correlated with the R2 parameter in both groups of subjects. The *r-value* for the whole group was 0.285, for women in the <40 group 0.330 (Figure 5).

**Figure 6:**
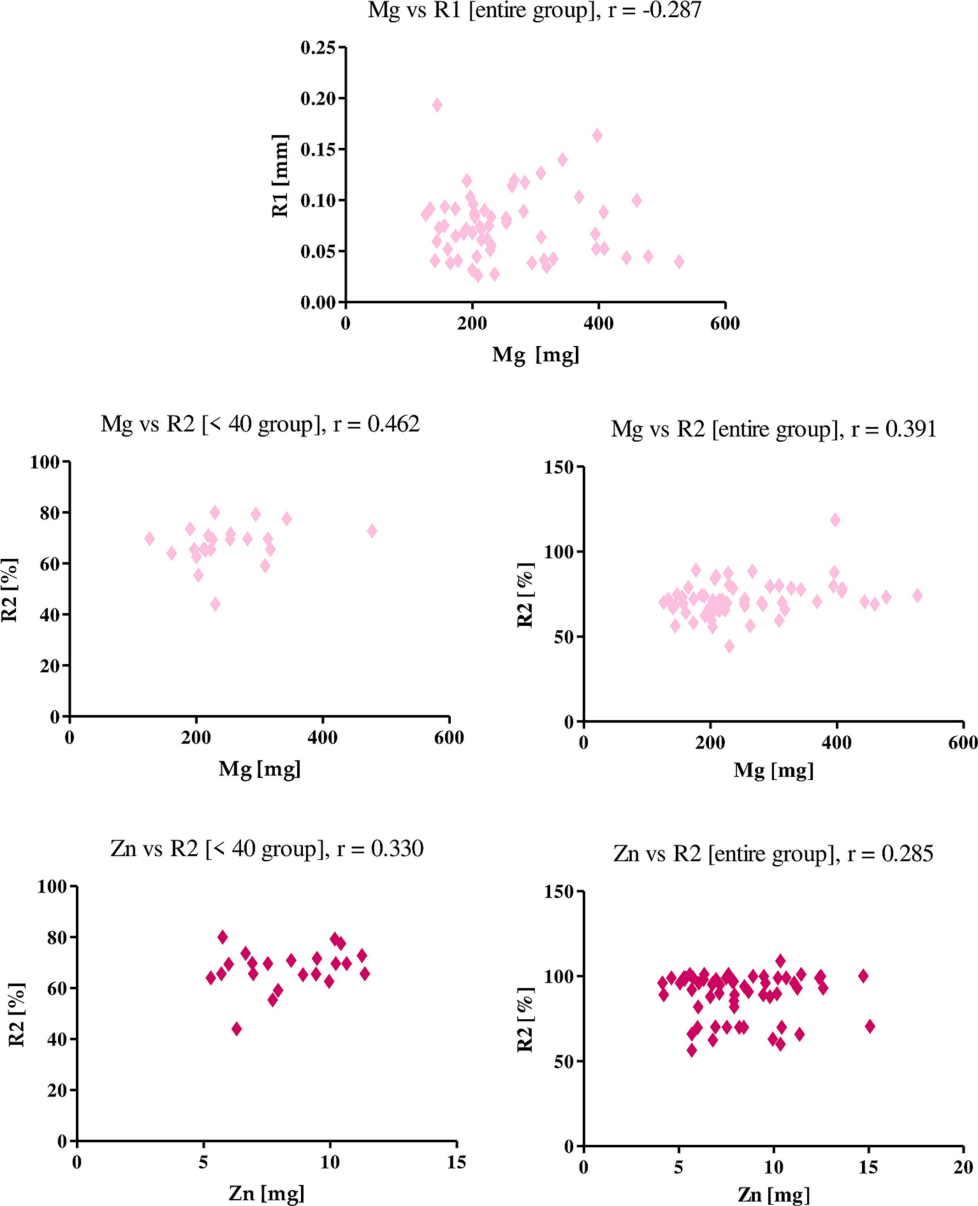
Correlations for copper with a reported *r* value.

Copper intake with diet showed a negative correlation with the parameter R1 (*r* = −0.364) in the entire study group, a positive correlation with the parameters R2 (in the entire group and age group <40) and R5 (in the entire study group; Figure 6). The *r-value* for the R2 parameter in each group was 0.328, 0.424, respectively, while for the R5 parameter, the value for all the women in the study was 0.262. This indicates that a higher intake of copper from the diet decreases the level of skin firmness, while it has a positive effect on skin elasticity.

**Figure 7:**
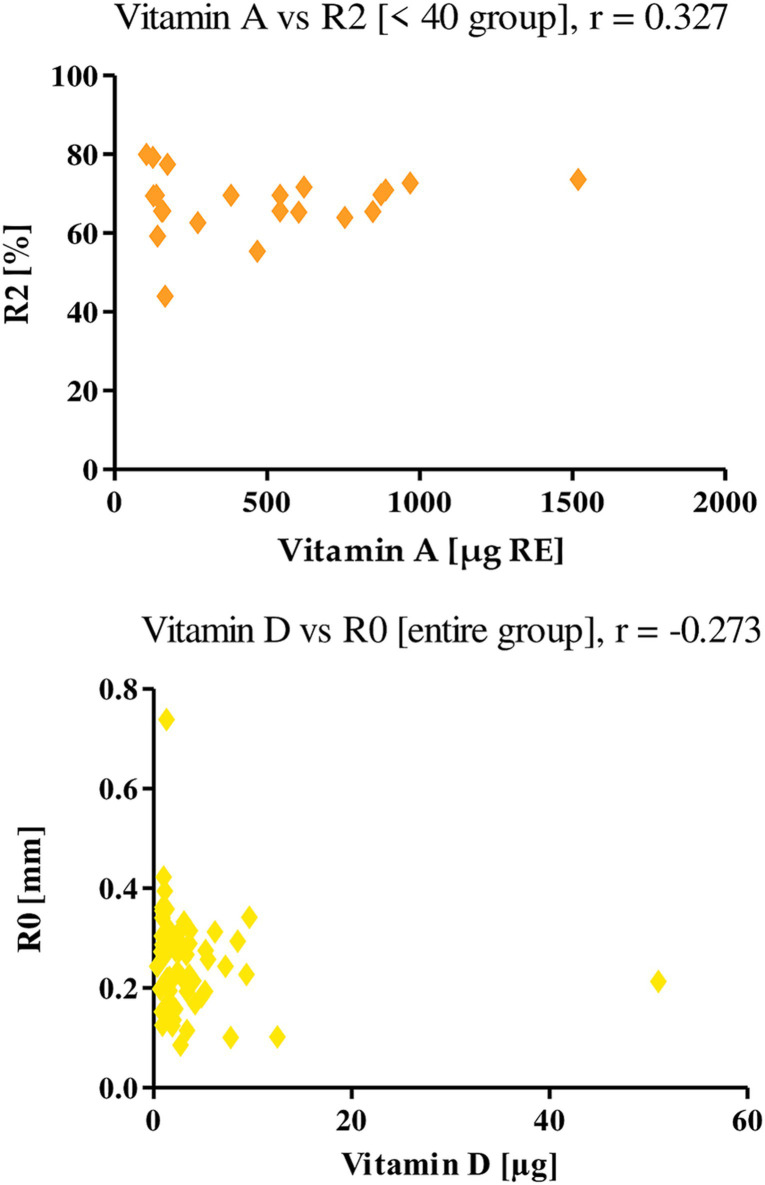
Correlations for vitamin A and vitamin D with a reported *r* value.

Vitamin A correlated only with the R2 parameter in the group of women under 40 years of age, r was equal to 0.327. This indicates that vitamin A consumed with the diet has a positive effect on the level of skin elasticity. Vitamin D, on the other hand, was the only one among the studied nutrients to correlate with the R0 parameter. The *r-value* was −0.273, indicating that the higher the intake of vitamin D, the degree of skin firmness among the studied women decreases (Figure 7).

**Figure 8:**
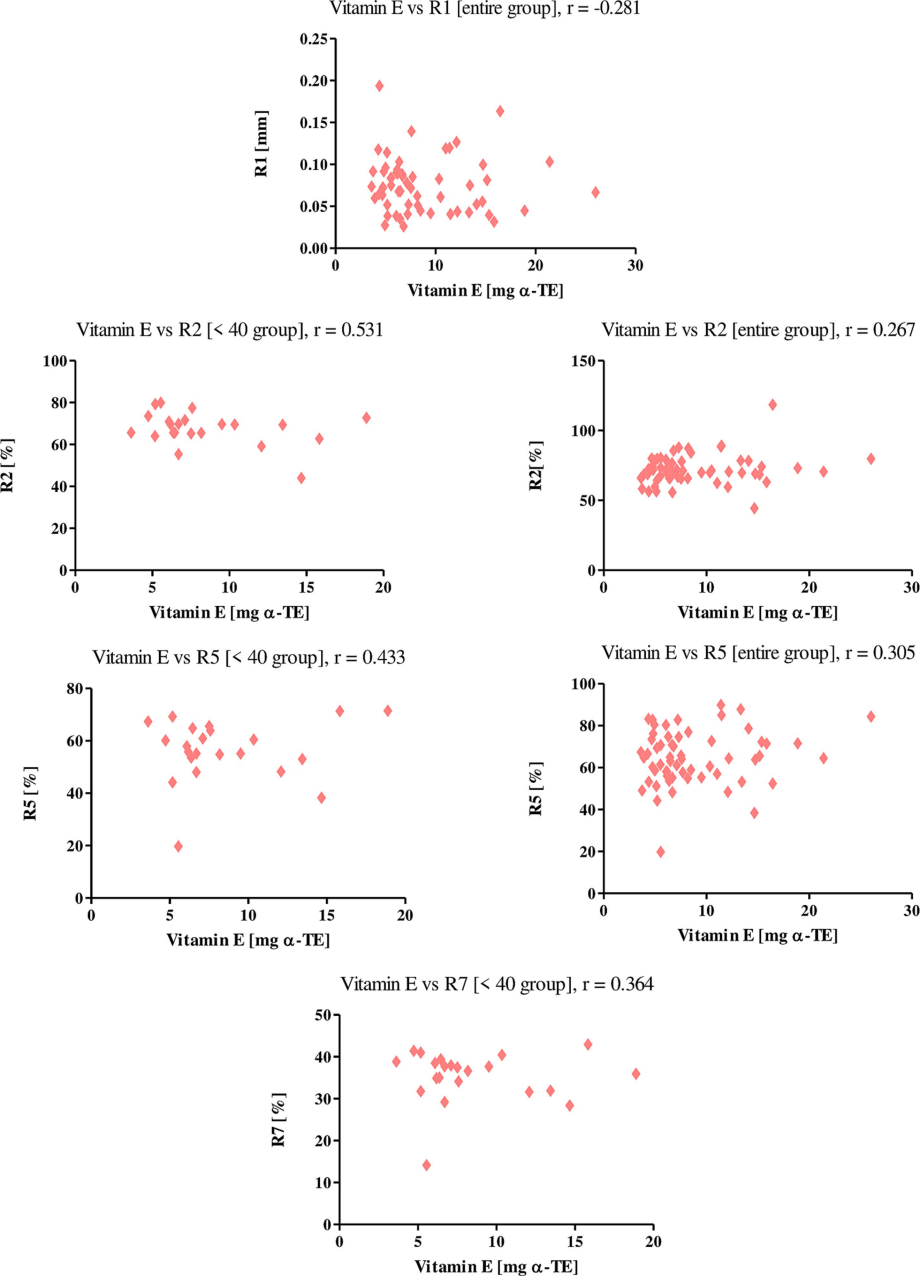
Correlations for vitamin E with a reported *r* value.

Vitamin E showed a negative correlation among the entire study group with parameter R1 (*r* = −0.281), indicating that skin firmness decreases as vitamin E intake with diet increases. With the parameters R2 and R5, on the other hand, vitamin E showed positive correlations, both among all women and those under 40 years of age. The r values for the R2 parameter were 0.267 and 0.531, respectively, and for the R5 parameter were 0.305 and 0.433, respectively. With the R7 parameter, vitamin E correlated positively only in the <40 age group, where the r was equal to 0.364 (Figure 8). This shows that an increase in vitamin E in the diet of the women studied increases the level of elasticity of their skin.

**Figure 9:**
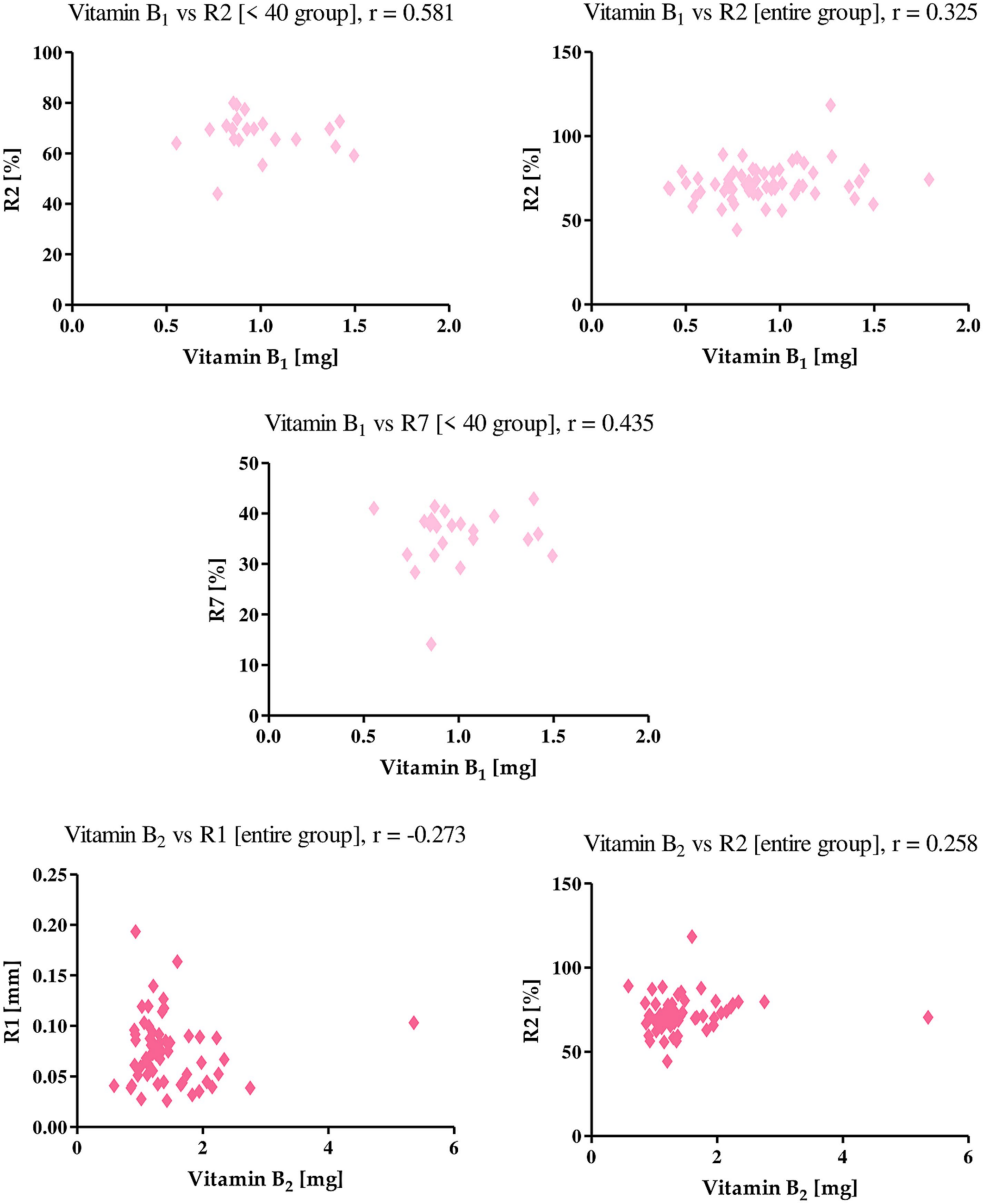
Correlations for vitamin B_1_ and vitamin B_2_ with a reported *r* value.

An increase in vitamin B1 intake positively influenced the level of skin elasticity among the women studied. This vitamin positively correlated with the R2 parameter in both the whole group (*r* = 0.325) and the <40 group (0.581), as well as with the R7 parameter in women under 40 (*r* = 0.435). Vitamin B2, on the other hand, showed a negative correlation with the R1 parameter and a positive correlation with the R2 parameter in the group of all women studied. The *r-value* was −0.273, 0.258, respectively, indicating that a higher intake of this vitamin along with diet decreases the level of firmness of women’s skin, however, it increases the level of elasticity (Figure 9).

**Figure 10:**
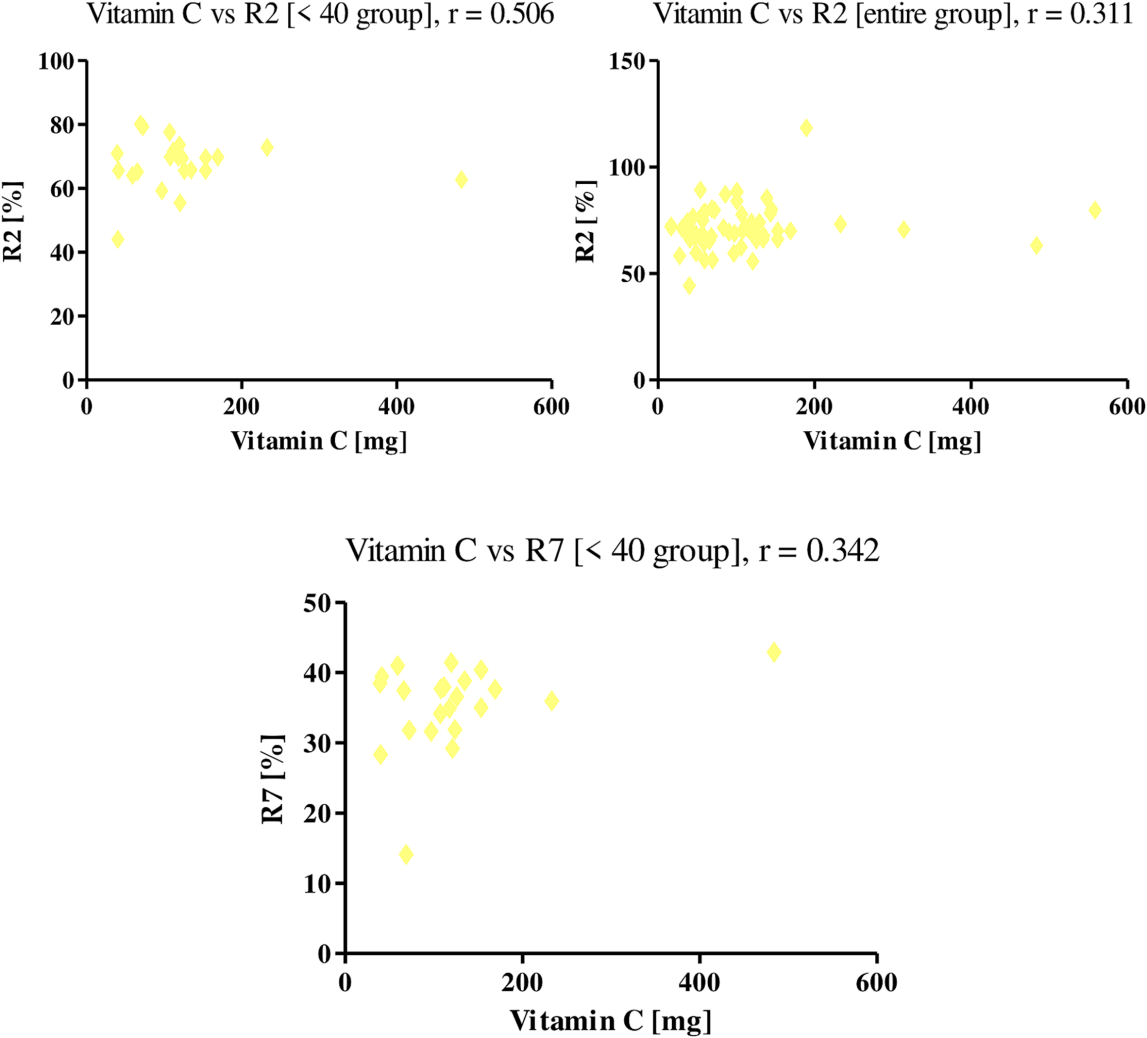
Correlations for vitamin B_6_ and vitamin B_12_ with a reported *r* value.

Vitamin B6 positively correlates with both the R2 and R7 parameter. The value of r in the correlation of vitamin B6 with the R2 parameter for the entire study group is 0.338, while for women in the <40 group, r takes the value of 0.381. With the R7 parameter, this component shows a correlation only in the group of women under 40, where r is equal to 0.360. This shows that with an increase in the level of vitamin B6 in the diet, the level of elasticity of the skin of the studied women increases. Vitamin B12, unlike vitamin B6, correlated only in the R1 parameter in the entire study group. The value of the correlation coefficient r was −0.261 (Figure 10). This means that an increase in intake with diet of vitamin B12 has a negative effect on skin firming.

**Figure 11:**
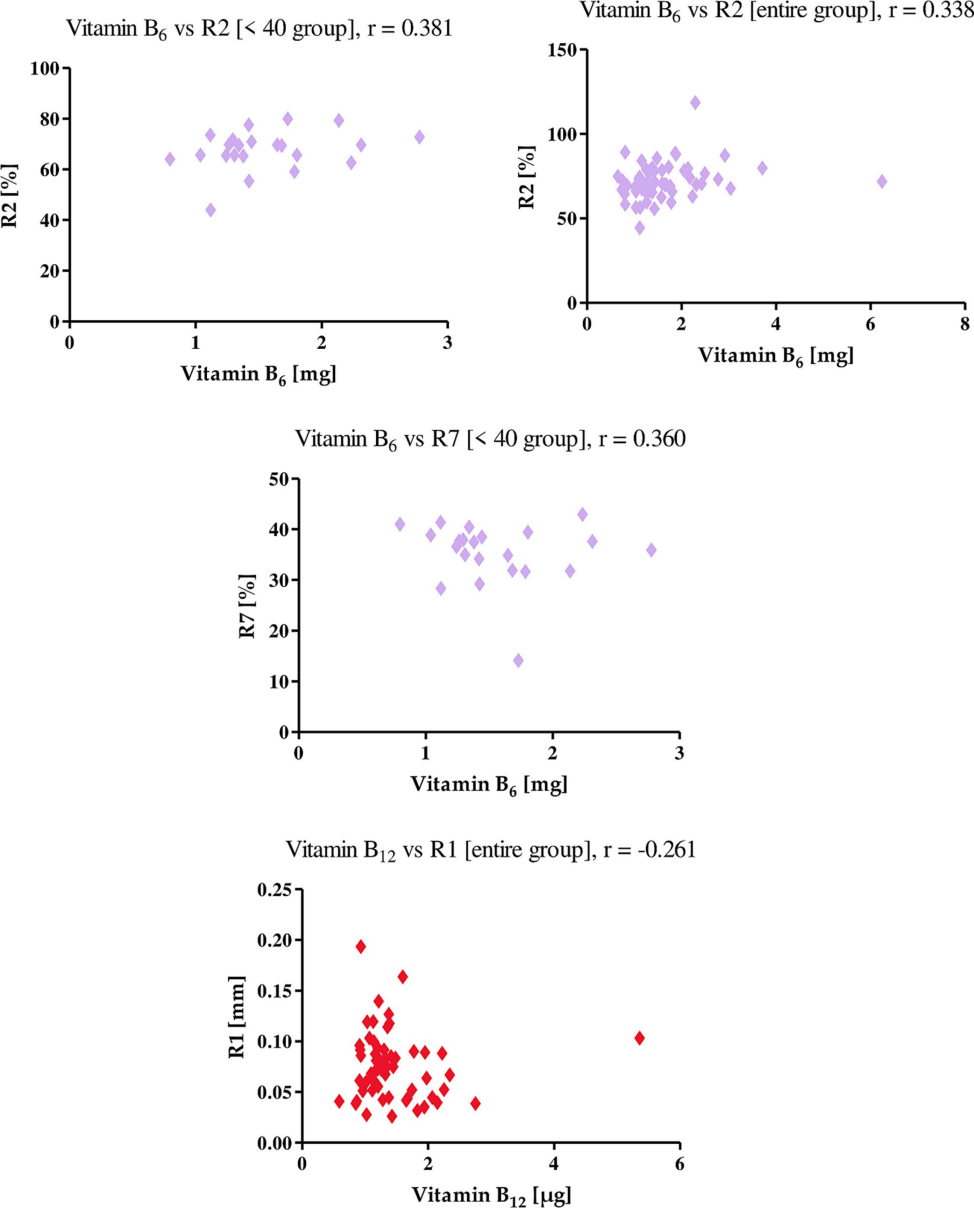
Correlations for vitamin C with a reported *r* value.

An increase in the intake of vitamin C with food positively affected the level of skin elasticity among the women studied. Vitamin C positively correlated with R2 and R7 parameters. The *r-value* for the R2 parameter among all women tested was 0.311, while in the group of women <40 r was equal to 0.506. With the R7 parameter, a correlation was shown only in the group under 40 (*r* = 0.342; Figure 11).

**Figure 12:**
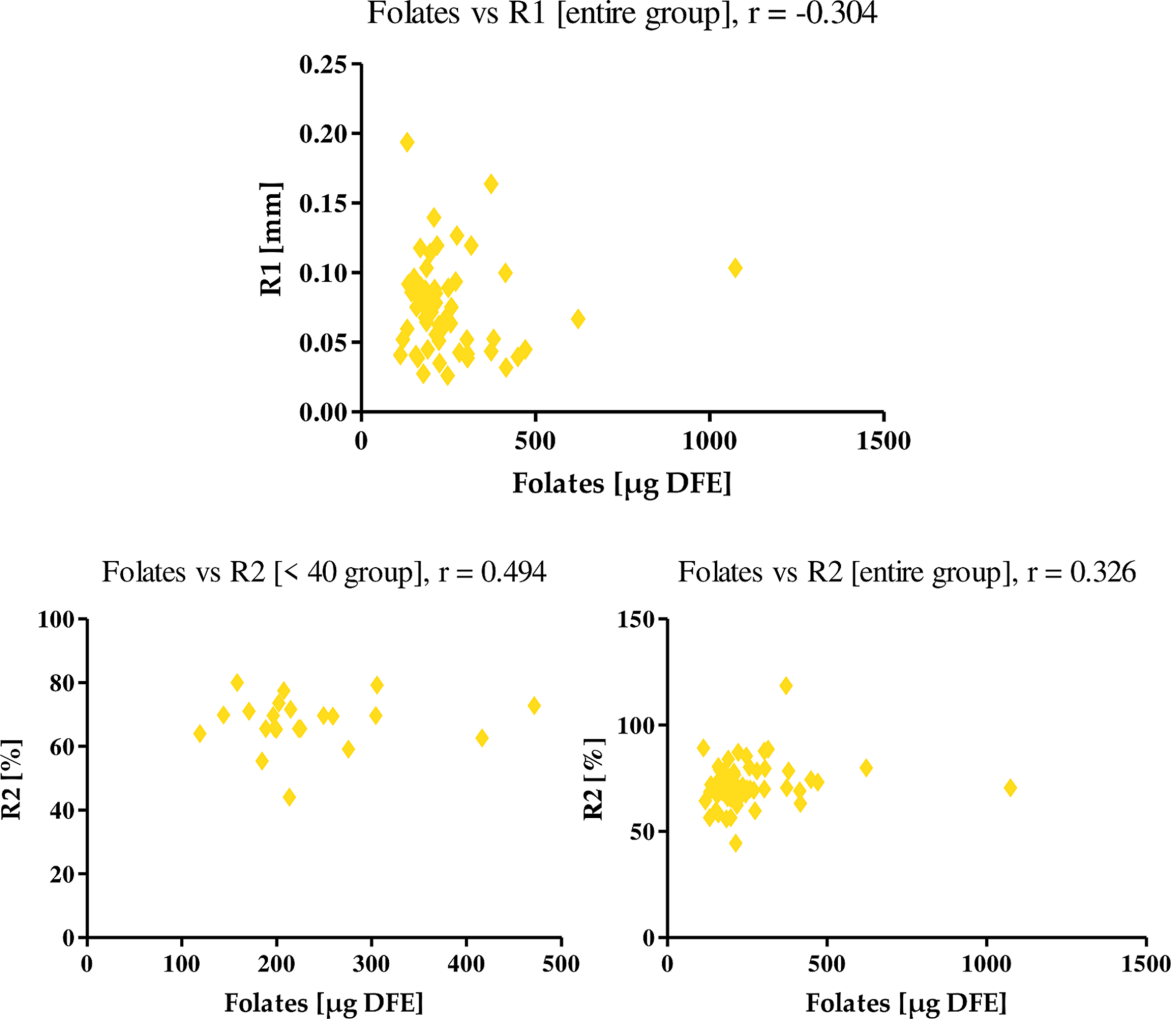
Correlations for folates with a reported *r* value.

The last nutrient under study, folate, negatively correlated with the R1 parameter in the entire group of women (*r* = −0.304) and positively with the R2 parameter, where r in the entire study group was 0.326, while among women <40, r was equal to 0.494 (Figure 12). This means that an increase in folate intake along with diet decreases firmness while increasing elasticity in women’s skin.

## 4 Discussion

The skin is a barrier separating the human body from the external environment hence the importance of keeping it intact, with the correct pH, proper hydration level and composition of the hydrolipidic layer (9, 22). A properly balanced diet is one of the factors supporting the maintenance of skin health. Inadequate nutritional status of the body negatively affects the integrity of the skin structure and its biological functions, which can result in the formation of skin barrier abnormalities and eventually translate into the formation of skin diseases or abnormalities such as acne, hyperpigmentation or wrinkles (6, 23–25). The skin exhibits numerous physical parameters, and among the most important are firmness and elasticity, which determine the appropriate strength of the skin’s coatings (26, 27). With this in mind, the purpose of this study is to evaluate the effects of selected nutrients on the level of elasticity and firmness of the skin. In our opinion, this is currently one of the first works to evaluate the role of dietary nutrients on skin function. In our study, we used non-invasive methods that did not disturb the integrity of the skin coverings. Because of this, the research done by our team can be repeated many times, by different research teams. We selected the most important nutrients for the study, however, only for some we found significant correlations. Accordingly, the discussion will refer only to the elements for which the aforementioned correlations were found.

Fatty acids can be synthesized in the skin *de novo* or can be uptaken with the diet, with the exception of short-chain polyunsaturated fatty acids (PUFAs), which are *α*-linolenic acid (ALA) and linoleic acid (LA). These acids cannot be synthesized in the skin, and thus must be obtained from food (28). To the best of our knowledge to date, we are the first research team to determine the effects of these acids on skin elasticity *in vitro*. In our study, we found a positive relationship between the amount of LA and ALA consumed with the diet and the level of skin elasticity. Interestingly, the Simard et al. team (28) found that supplementation with LA and ALA acids was associated with an increase in the absorption of testosterone through the skin, which translated into improved skin barrier function. This indicates that LA and ALA acids, should be provided in optimal amounts in the diet which will translate into improved skin quality. Foods rich in these acids include walnuts and flaxseed, as well as vegetable oils, eggs and milk (29, 30).

Another important relationship found in our study is the effect of dietary fiber on skin properties. It is unfortunate that we are the first research team to find a similar relationship, so we are unable to relate our results to the work of other teams. According to most studies, daily consumption of dietary fiber has a beneficial effect on the normal functioning of the human body, however, its exact role on skin function is currently unknown. An important property of fiber is the regulation of the absorption of trace elements such as iron (31). The normal concentration of iron in the blood depends on absorption in the gastrointestinal tract and its normal concentrations are closely related to normal skin function (32). According to a few studies, fiber has an effect on wound healing (33). Taking into account the above and the reports obtained from our research, it seems expedient to provide plenty of dietary fiber with the diet. Among its best sources are groats and products of plant origin (31).

The study also showed a correlation between the content of trace elements and micronutrients in the diet and skin properties. Our research has shown that the dietary content of Na, K, Mg, Zn, Cu has a significant effect on the elastic properties of the skin. All of the aforementioned elements play an important role in the normal functioning of the body, but are also involved in the physiological functions of the skin (32). Although these elements show positive effects on skin elasticity, excessive supplementation of these elements in the diet is not advisable. Excessive supplementation of trace elements has been shown to translate into the possible development of diseases such as hypertension (excessive sodium supply) (34). Importantly, also in the course of skin diseases such as psoriasis and acne, abnormal levels of trace elements are found, indicating that they may be involved in the development of skin disorders (33). Therefore, when deciding whether to supplement or expand the diet with the mentioned micronutrients, it seems advisable to first perform medical, laboratory and dermatological tests.

B vitamins, are now widely available in supplements; however, their exact role in skin physiology is currently poorly understood. Deficiencies of vitamins from this group are associated with skin diseases such as seborrheic dermatitis, atopic dermatitis and acne (35). According to the results we obtained, a diet rich in vitamins from these groups has a beneficial effect on skin parameters. Importantly, the introduction of a diet rich in these vitamins will be associated not only with an improvement in skin parameters, but also with a decrease in the risk of contracting certain dermatoses. However, it is important to note that some B vitamins may increase the severity of existing skin diseases (35). Therefore, as with enriching the diet with more micronutrients, a dermatologist should be consulted beforehand.

The skin is an organ that has a high demand for folate - this is mainly due to its regenerative properties. In addition, folate has a significant effect on skin cell metabolism and has a protective effect on skin photoaging. Importantly, skin cells have been shown to increase folate uptake when exposed to UV radiation (36). In light of our research, and given that folate plays an important role in skin protection, it is advisable to enrich the diet with this nutrient.

In the case of vitamin E, dermatological research on this vitamin has been ongoing for many years. Despite this, there is currently a lack of clinical studies determining the appropriate dose and clinical indications for supplementation of this vitamin. The most important function of vitamin E is antioxidant activity, which protects the skin from free radicals. Importantly, even with high doses, few people report any side effects from taking this ingredient (37). Considering this fact and the results obtained from our study, it is advisable to enrich the diet with products containing vitamin E.

The skin is the site of vitamin D synthesis, in addition, this element has an extremely important role in skin physiology. Vitamin D affects keratinocyte proliferation and differentiation, induction of apoptosis and control of the immune response. According to most studies, vitamin D supplementation and the use of a vitamin-rich diet have beneficial effects on the skin in the course of skin disorders such as psoriasis and acne (38). In light of our study, vitamin D has an effect on skin firmness. This is an important report that indicates that a vitamin D-rich diet can have a beneficial effect on skin parameters.

The last compound that influenced skin parameters in our study was vitamin C. This vitamin, like vitamin E, is a powerful antioxidant. In addition, this vitamin is necessary to stimulates collagen synthesis and also protects the skin from photoaging (39). This translates into the results we obtained, as we found a positive effect of a vitamin C-rich diet on skin properties.

In conclusion, our research indicates that certain nutritional elements have a positive effect on skin properties. Therefore, enriching the diet with the compounds we described will translate into improved properties of this organ. However, it is important to keep in mind that some of the components we have described may have an adverse effect on existing skin diseases, and their excess may be associated with the development of diseases. Therefore, before making dietary modifications, consult a nutritionist, doctor or cosmetologist.

At the same time, our work has some limitations. First, the number and gender of patients. We selected only women for the group, in future studies we also want to study the effect of diet on the skin condition of men. It is unfortunate that our study includes a small number of female patients. In future studies, we plan to increase the number of female subjects to 100. In addition, we were limited by the ratio of women before the age of 40 to women over 40. In future studies, the numbers of both groups will be equal.

Secondly, the selection of the research method. In our study, we used only one research method. In the future, we plan to expand our study to include other research methods.

Apparent limitations aside, our work is groundbreaking because we are the first team to determine the role of nutrients on skin parameters.

## 5 Conclusion

Thiamine, Riboflavin, Folate, Vitamins B6, B12 and C correlated with skin elasticity. Higher correlation coefficients were found in the group of women <40 years old than in the total group (21–74 years old). Of the nutrients studied, only vitamin D had an effect on the parameter reflecting skin firmness. Low intake of LA and ALA acids, dietary fiber, as well as potassium, vitamin D and folate were found in the group of women studied. The collected studies indicate that providing adequate nutrients with the diet has a positive effect on the proper functioning of the skin. The results of our research can be helpful in composing supplementation to maintain healthy skin. In addition, it is a good tool to combine the work of a cosmetologist, dermatologist and nutritionist to obtain visible and beneficial changes in skin quality.

## Data Availability

The raw data supporting the conclusions of this article will be made available by the authors, without undue reservation.
